# Editorial: Harnessing the potential of duckweed: biological insights and ecological applications

**DOI:** 10.3389/fpls.2026.1887238

**Published:** 2026-06-30

**Authors:** Zhihong Xu, Hai Zhao, Shunong Bai, Eric Lam, Lin Yang

**Affiliations:** 1 School of Life Science, Peking University, Beijing, China; 2 Chengdu Institute of Biology, Chinese Academy of Sciences, Chengdu, China; 3 Department of Plant Biology, Rutgers the State University of New Jersey, New Brunswick, NJ, United States; 4 Tianjin Key Laboratory of Animal and Plant Resistance, College of Life Sciences, Tianjin Normal University, Tianjin, China

**Keywords:** application, bioreactor, duckweed, new resources, stress response

The growing human population has increased the need for improved food security, nutritional adequacy, and environmental sustainability. The global food system remains heavily reliant on a narrow spectrum of staple crops and faces mounting environmental pressures. Many conventional crops have a harvest index generally between 0.3 to 0.5, meaning that only a fraction of the total biomass produced can be efficiently recovered for industrial or commercial use. This creates significant challenges for future agricultural and resource systems. Duckweed (*Lemnaceae*) shows highly efficient photosynthesis capability and has one of the fastest biomass accumulation rate known among flowering plants (Acosta et al., 2021).

**Figure 1:**
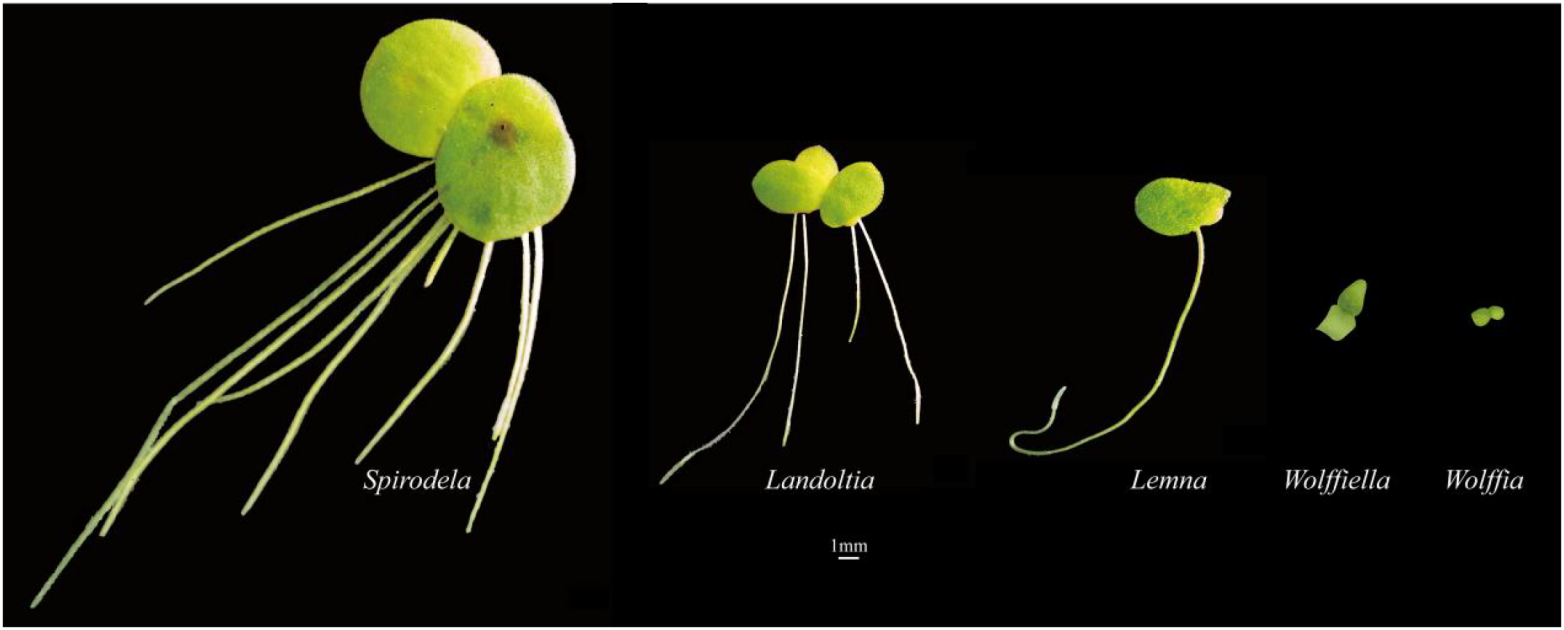
The 5 genera of duckweed: *Spirodela*, *Landoltia*, *Lemna*, *Wolffia*, and *Wolffiella*.

As the smallest flowering plants on Earth, duckweed possess an outsized potential for both fundamental research and applied biotechnology. Comprising of five genera (*Spirodela*, *Landoltia*, *Lemna*, *Wolffiella*, and *Wolffia*) (**Figure 1**), these aquatic angiosperms exhibit the fastest growth rates among flowering plants, clonal reproduction, and remarkable adaptability to diverse environments. In general, they maintain continuous clonal growth and can double in mass or number every 24-48 hours (Kim et al., 2024). In many tropical or sub-tropical regions, duckweeds are used as traditional feed or are even sold as vegetables in local markets (Mes et al., 2022). Under specific stress conditions, duckweed can quickly convert assimilates into storage compounds in major plant tissues (Liu et al., 2015). Duckweeds have recently gained attention as promising candidates for sustainable agriculture, wastewater remediation, and bio-manufacturing. .

Driven by advances in genomics, genetic transformation, controlled-environment agriculture, and food technology, duckweed research has entered a new era. This Research Topic, “Harnessing the Potential of Duckweed: Biological Insights and Ecological Applications” brings together cutting-edge studies that explore the functions of duckweed ecological stress responses, and applications in nutrient recovery and high-value compound production.

Over the past decades, efforts have been devoted to developing duckweeds as alternative crops for food security and as bioreactors for high-value products (Kaplan et al., 2019; Chen et al., 2022). Clonal growth allows duckweed to rapidly spread across the water surface and maximaze photosynthetic area in two dimensions. When combined with appropriate culture systems, this unique growth pattern gives duckweed an exceptional potential to maximize its productivity by expanding growth space without reducing photosynthetic efficiency. Given the urgent need for alternative crops that can help tackle challenges such as climate change and population growth, duckweed has substantial potential to improve the production of various nutrients and plant products (Acosta et al., 2021). A simple cultivation strategy combining nutrient limitation and elevated CO_2_ can dramatically increase duckweed (*Landoltia punctata*) starch content to 72.2% (dry mass basis), with a productivity equivalent to 38.0 t ha^-1^yr^-1^, surpassing that of traditional starch crops has been reported in this issue. (https://doi.org/10.3389/fpls.2025.1531849). This shows how simple environmental cues can redirect plant metabolism and provide a low-tech but high-impact solution to global starch demand.

Beyond biomass production, duckweed also contributes to sustainable agriculture and environmental protection. Sasmaz Kislioglu et al. demonstrated that the common duckweed (*Lemna minor*) can efficiently remediate meat-processing wastewater by purifying water and capturing nutrients into harvestable biomass (https://doi.org/10.3389/fenvs.2025.1622266). Yadav et al. further explored the optimization of biomass quality through organic fertilization, which showed cow dung application affected both biomass yield and the accumulation of bioactive compounds.

The establishment of duckweed as a tractable model system for plant biology has been driven by progress in genome sequencing and genetic transformation. High-quality, chromosome-scale genome assemblies are now available for multiple species across the *Lemnaceae* family. Notably, the genomes of *Spirodela polyrhiza* (An et al., 2019), *Landotia punctata* (Fang et al., 2025), *Lemna minor*, *Lemna turionifera*, *Lemna gibba*, and *Wolffia australiana* (Li et al., 2023) have been resolved at the chromosome level, with recent work further assembling subgenomes of the interspecific hybrid *Lemna japonica* (Ernst et al., 2025). Furthermore, the plant-on-chip culture system has been applied, enabling long-term observation and experimental manipulation within very small volumes, which not only facilitates high-resolution tracking of morphogenesis but also redefines plant research paradigms by integrating miniaturized culture platforms (Li et al., 2023). These resources have enabled comparative genomic analyses of gene losses and gains associated with reduced morphology, clonal reproduction, duckweed evolution (Fang et al., 2025), and unique epigenetic regulation. In parallel, efficient and reproducible genetic transformation protocols have been established for several duckweed species, including *Lemna minor* (Yang et al., 2018), *Lemna gibba* (Yamamoto et al. 2001), and the highly transformable *Lemna japonica* accession. *Lemna japonica* 8627, in particular, has been developed as a robust platform for recombinant protein expression and metabolic engineering because of its reduced epigenetic silencing and efficient regeneration from tissue culture (Ernst et al., 2025). Together, these advances have transformed duckweed from a physiological curiosity into a genetically accessible platform for both basic research (Yang et al., 2023) and biotechnology applications. Over the past decade amongst the top five institutions contributing to duckweed research, two are based in China and three are based elsewhere, with the Chinese Academy of Sciences (Zhao Hai group) ranks first in publication volume, followed by Rutgers University and Tianjin Normal University.

These advances enable precise genetic engineering and support mechanistic studies of development and stress responses. Using *Lemna turionifera* 5511, Di et al. investigated the role of tryptamine in regulating plant growth, senescence, and stress responses. This study found elevated endogenous tryptamine levels in senescent duckweed, suggesting a possible association between tryptamine accumulation and aging (https://doi.org/10.3389/fpls.2025.1625939). Qu et al. examined the role of the neurotransmitter moleculeγ-aminobutyric acid (GABA) in modulating Cd stress response and tolerance in duckweed. Overexpression of glutamate decarboxylase (GAD), a key enzyme catalyzing the conversion of glutamate (Glu) to GABA, enhanced Cd tolerance.

Duckweed has also been used as a sensitive and versatile model for assessing emerging environmental contaminants. Gjata et al. provided a systematic comparative analysis of the phytotoxicity of seven rare earth elements (REEs), showing that holmium and lutetium exert the most severe toxic effects while cerium induces a distinct hormetic response. In parallel, Boldrini et al. demonstrated the adaptive capacity of duckweed under chronic low-dose-rate radiation stress, identifying phase-specific acclimation strategies involving morphological adjustments, photosynthetic pigment modulation, antioxidant responses, and epigenetic regulation.

Together, these contributions validate duckweed as a powerful bioassay organism for environmental monitoring, complex physiological studies, and investigation of molecular mechanisms underlying its resilience to anthropogenic stressors. This applied value is supported by genome decoding and genetic tool development, which continue to expand the potential of duckweed in basic biology, environmental applications, and synthetic biology.
